# My Fellow-Probationer

**Published:** 1918-11-23

**Authors:** 


### SOME HOSPITAL TYPES OF BRITISH NURSING.

### My Fellow-Probationer.

We were both very tired probationers working in a heavy Male Surgical Ward somewhere in London long ago, a ward possessing a feeble Sister, and a shrewd dominant Staff Nurse who made the days harder, and more difficult to bear.

There was so much to remember—so much to do, and our Brains were dulled by the strangeness of it all—and our Muscles almost inert from over-exertion—and behind us was Staff Nurse impossible to satisfy.

I admit to a sincere hatred of this Nurse, but my fellow-probationer—little Nurse Helen—fair-haired, blue-eyed—and always so neat and clean-looking, seemed able to bear patiently all the snubs and oppression. I, with less patience, marvelled at this Nurse Helen. She was of the Upper Servant Class—not Book educated—but as courteous and well-mannered as (I will not say—a Lady) but as a Gentlewoman—from the first a nurse.

I envied the adaptability and tenderness which made her such a favourite with the Patients—and it went to my Heart when a poor Spinal Case on my side of the ward said—"I wish Nurse Helen might lift me—she don't hurt at all." She was always ready to lend a helping hand—to fill a Hot Water Bottle or give a Drink. "It isn't my work" was no word of hers. It was all one to her—Senior or Junior Duties, she was ready to do the thing to hand ungrudgingly. I believe she would have taken Night Duty as well as Day if allowed—and "Off Duty" seemed little joy to her.

She was thorough and loved nursing. Even Staff Nurse allowed she kept the Boys and old Men "Smart." A merry little probationer too, with a vein of cheerfulness that was contagious.

One night going "Off Duty" with her I relieved my wrath by wishing punishment to our Staff Nurse. Nurse Helen said—"I do not feel angry—I shall tell all to my Heavenly Father."

Yet she was no prig—or over-righteous girl—just a born nurse with a big Heart for others. Not theoretically clever—but clever with the fine natural power sometimes seen in what is called the lower class.

As a probationer—obedient, gentle, skilful, with hands made for sore places, her bright face bending over a patient, ready for extra service, too often passed over by others, what wonder that she became one of the most trusted and beloved Staff Nurses in the Building?

☛ For "Should the Nurse Articulate?" by "Ierne," see p. 162; "Is Every Woman a Nurse?" p. 169 Each British Woman to be Stirred Up? p. 169; College Meetings and Fixtures, p. 170.

